# Advancing digital health in China: Aligning challenges, opportunities, and solutions with the Global Initiative on Digital Health (GIDH)

**DOI:** 10.1002/hcs2.118

**Published:** 2024-10-17

**Authors:** Ge Wu, Mengchun Gong, You Wu, Li Liu, Boyang Shi, Zhirong Zeng

**Affiliations:** ^1^ Multi‐modal Data Application Lab Guangdong Medical University Zhanjiang 524003 China; ^2^ National Clinical Research Center for Aging Fudan University Huashan Hospital Shanghai 200040 China; ^3^ Institute for Hospital Management, Tsinghua Medicine Tsinghua University Beijing 100084 China; ^4^ Department of Health Policy and Management, Bloomberg School of Public Health Johns Hopkins University Baltimore 21205 MD USA; ^5^ Department of Health Management Nanfang Hospital Guangzhou 510515 China

**Keywords:** Artificial intelligence, Data Standards, Digital health, Multi‐modality data

## Abstract

We summarized the unique challenges that China faced in digital health due to its large population, regional disparities, and uneven distribution of medical resources. Under the guidance of the Global Initiative on Digital Health (GIDH) released by WHO, we proposed corresponding solutions that address infrastructure, data, terminology, technology and security.

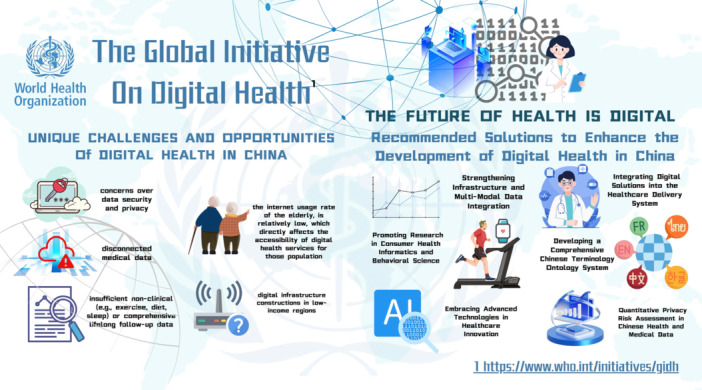

**Abbreviations**
CMeSH: Chinese Medical Subject Headings
EMR: electronic medical record
GIDH: Global Initiative on Digital Health
HIPAA: Health Insurance Portability and Accountability Act
LOINC: logical observation identifiers names and codes
LLM: Large Language Models
SNOMED CT: systematized nomenclature of medicine ‐ clinical terms
TCMLS: Traditional Chinese Medicine Language System

## 1 THE FUTURE OF HEALTH IS DIGITAL

**Figure 1:**
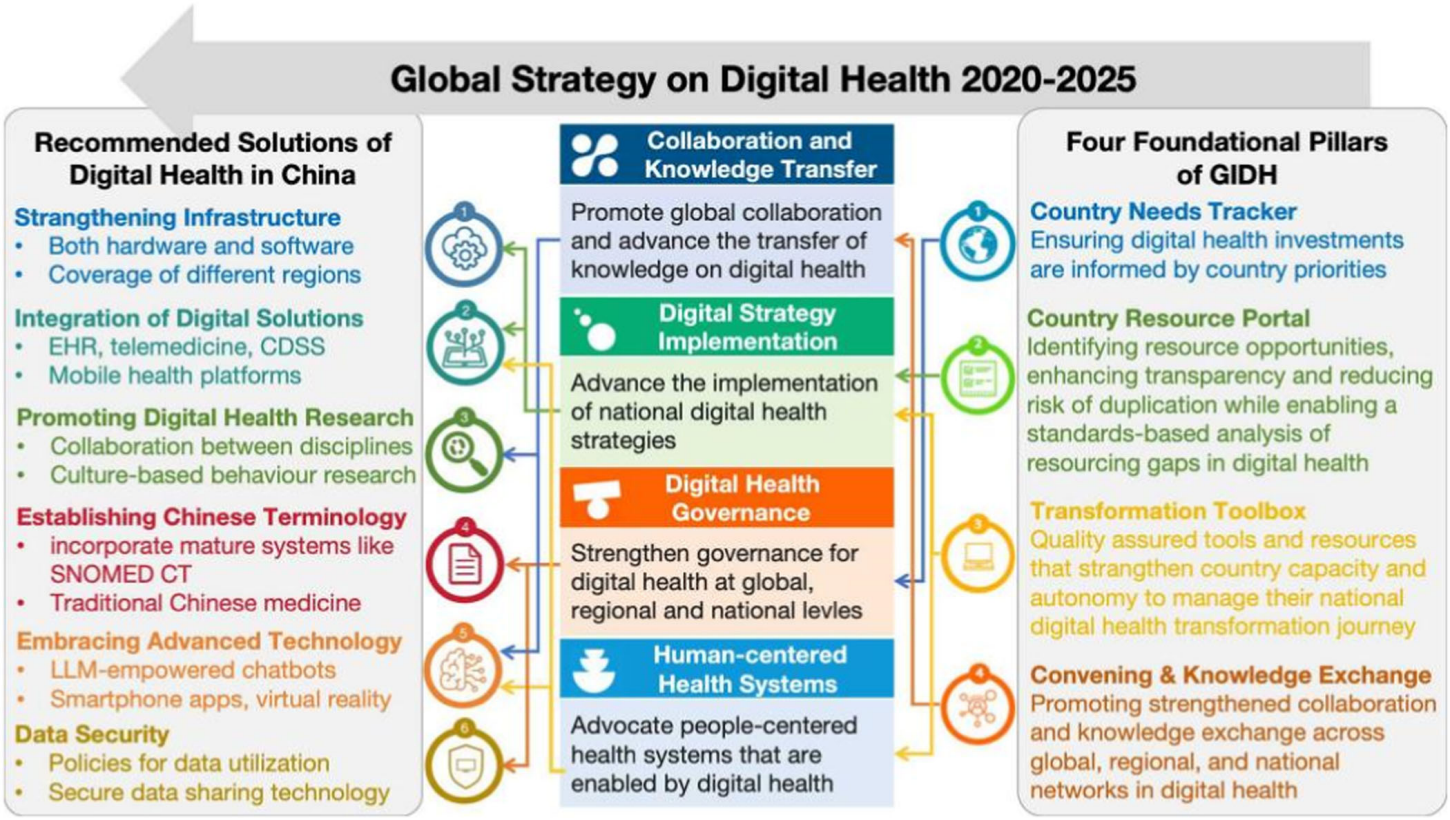
The four foundational pillars of GIDH that supports the implementation of WHO Global Strategy on Digital Health 2020‐2025 and recommended solutions of digital health in China.

Digital health has shown significant promise in improving health outcomes. However, its transformation faces various challenges, including resource distribution disparities across countries, varying definitions and standards for digital solutions, and a lack of coordination in digital health investments [1]. To address these issues, the Global Initiative on Digital Health (GIDH) has identified four essential pillars: national‐informed digital health investments, a transparent national resource portal, a transformation toolbox for management, and facilitating knowledge exchange at national, regional, and global levels. GIDH, as a WHO Managed Network, is committed to supporting the *Global Strategy on Digital Health 2020–2025*, promoting national digital health transformation through technical support, addressing resource and financial gaps, regularly updating tools, and fostering cross‐network knowledge sharing (Figure 1).

## 2 UNIQUE CHALLENGES AND OPPORTUNITIES OF DIGITAL HEALTH IN CHINA

Digital health has demonstrated its capability in both clinical interventions and policy support [2]. In recent years, China has made notable advancements in this field. According to the “2024 China Digital Healthcare Industry Market Outlook Forecast Report” by the China Academy of Commerce Industry Research, China's digital healthcare market reached 195.4 billion CNY in 2022, with an average annual growth rate of 30% over the last five years. Moreover, 125 national regional medical centers have been established in second‐ or third‐tier cities. By 2022, electronic medical record (EMR) coverage will reach 90% in tertiary hospitals, 60% in secondary hospitals, and 40% in primary hospitals. Over 3,000 internet hospitals have been established, and telemedicine services now cover city and county levels, benefiting more than 25.9 million people. For special populations such as the elderly, developers have created simpler app modes with larger fonts to enhance accessibility.

Despite these advancements, China faces unique challenges in digital health due to its large population, regional disparities, and uneven distribution of medical resources. Some of the challenges include:

a. Digital infrastructure in low‐income regions: Network infrastructure construction and digital medical penetration rates remain relatively low in rural areas, directly impacting the accessibility of digital health services.
b. Disconnected medical data: System incompatibility and inconsistent data standards limits patient medical data within individual hospitals, forming “information silos”. There are reportedly thousands of different medical information systems across China, hindering data exchange and sharing between systems.
c. Insufficient nonclinical: China's healthcare system lacks robust mechanisms for collecting and analyzing nonclinical data (e.g., exercise, diet, sleep) or conducting comprehensive lifelong follow‐ups. While health management apps are ganing popularity, overall usage remains relatively low.
d. Accessibility for various populations: according to the China Internet Network Information Center, internet usage among the elderly, especially in rural areas, is relatively low, limiting their access to digital health services.
e. Data security and privacy concerns: Frequent data breaches in China's medical field have raised privacy concerns. Over 80% of respondents in market research expressed worries about medical data security, and privacy protection policies are still incompatible.

Despite these obstacles, significant strides have been made in information system development in recent years, particularly during the COVID‐19 pandemic, with digital technologies enhancing surveillance, decision support, follow‐up care, and access to non‐COVID‐19 medical services [3]. Nonetheless, the effective implementation of digital health initiatives is hindered by persisting issues related to data standards and sharing, particularly with the growing diversity of inter‐institution data sharing, multi‐modal medical data, and the need for high‐performance infrastructure and AI technology.

Challenges often bring opportunities, and as China continues to implement the “Healthy China 2030” plan for over five years, the establishment of a robust health information service system has emerged as a critical priority. However, the development of digital health in China still has a long way to go. Aligned with the GIDH guide and the specific national context, we propose the following recommendations to guide the development and implementation of digital health initiatives in China.

## 3 RECOMMENDED SOLUTIONS TO ENHANCE THE DEVELOPMENT OF DIGITAL HEALTH IN CHINA

In response to the challenges mentioned above, we propose six goals that address infrastructure, data, terminology, technology, and security. These provide guidance for the future development of digital health in China. However, due to disparities in development and variations in policy factors, there may be discrepancies in the level of digital health infrastructure across different regions, and even among different hospitals within the same city. Specific implementation for different medical institutions must take into account various factors, including infrastructure development, budgetary constraints, computational requirements, public demand, and local policies.

### 3.1 Strengthening infrastructure and multi‐modal data integration

The development of digital health critically hinges on bolstering information infrastructure, particularly technology‐based digital systems. This involves fostering the expansion, collaborative sharing, and intelligent upgrading of information infrastructure. While China has made notable strides in digital construction, there remain considerable unmet needs of over one‐third of the population residing in rural areas, who may face limitations due to educational levels, economic conditions, or lack of internet access [4]. Therefore, prioritizing infrastructure enhancement is imperative for the upcoming decade. This should encompass hardware such as network facilities, medical equipment (e.g., IoT), information security hardware, and software such as medical information systems, big data, AI applications, and mobile apps. This development will create significant demand for suppliers. By 2024, the market size of China's digital healthcare is expected to expand to 413 billion CNY, more than doubling from 2022. With this huge market, government and healthcare institutions need to conduct long‐term planning and establish supervision and management systems to avoid resource waste.

Advancements in AI technology have significantly improved the processing and analysis of multi‐modal data. Research has shown that integrating radiologic and histologic features with clinical and genomic data enhances cancer patient risk stratification [5]. The integration and governance of multi‐modal data hold substantial value in digital health, as they can enhance diagnostic accuracy, optimize resource allocation, and foster advancements in medical research. With the continuous progression of technology and expanding application scenarios, the potential applications of multi‐modal data in digital healthcare are set to increase.

### 3.2 Integrating digital solutions into the healthcare delivery system

The GIDH underscores the importance of leveraging digital innovations to enhance healthcare accessibility, efficiency, and quality across the continuum of care. Integrating digital solutions into the entire healthcare delivery system is an essential strategy for transforming health services in China. This involves extending beyond traditional hospital care to encompass out‐of‐hospital services facilitated by digital technologies [6]. Electronic health records, telemedicine, AI‐driven diagnostics, and mobile health platforms, when incorporated across all aspects of healthcare delivery, have the potential to enable seamless patient data flow, support real‐time decision‐making, and facilitate personalized care plans. Thus, in turn, can improve health outcomes and enhance patient experiences.

### 3.3 Promoting research in consumer health informatics and behavioral science

The utilization of digital health for delivering behavioral interventions related to physical activity, diet, drug use, and mental health is increasing [7]. However, China's diverse regional cultures and customs necessitate research on the application and effectiveness of these digital health interventions in various cultural contexts. Developing health intervention methods that are tailored to the unique needs of Chinese consumers in different regions is essential. Additionally, fostering collaboration between disciplines such as medicine, informatics, and psychology will be crucial for advancing research in health informatics and behavioral science. Interdisciplinary cooperation will help to better understand and address the cultural nuances that influence the effectiveness of digital health interventions in China.

### 3.4 Developing a comprehensive Chinese terminology ontology system

The development of a widely adopted Chinese terminology ontology system is crucial for ensuring interoperability and supporting data mining efforts. Although digital health technology adoption increased during the COVID‐19 pandemic, it fell short of expectations partly due to the lack of standardized data [8]. Most existing medical terminology systems are designed for English speakers, and the current Chinese clinical medical terminologies – such as the Chinese version of ICD‐10, Chinese Medical Subject Headings (CMeSH), and the Traditional Chinese Medicine Language System (TCMLS) – primarily consist of medical vocabularies. However, these terminologies exhibit weak semantic and associative relationships and lack a dynamic updating mechanism.

To address these issues, the design of a Chinese medical terminology/ontology system should learn experience from the mature systems such as SNOMED CT and LOINC, along with a continuously updated database and corpus for standardizing clinical data. Furthermore, traditional Chinese medicine terminology must also be standardized. With the support of this terminology ontology system, natural language processing and deep learning technologies can be applied to extract valuable information from the vast amount of clinical data, advancing the capabilities of digital health technologies in China.

### 3.5 Embracing advanced technologies in healthcare innovation

As we enter the era of AI, the widespread use of Large Language Models (LLM) is becoming increasingly inevitable. The success of ChatGPT in demonstrating the capabilities of LLM‐empowered chatbots highlights their potential to revolutionize the healthcare industry [9]. Therefore, it is crucial to proactively explore the applications and viability of integrating chatbots across various healthcare sectors. Although access to ChatGPT is restricted in China, many companies and universities are researching and developing their own medical LLM. Additionally, emerging technologies such as smartphone apps, virtual reality, and social media are reshaping healthcare practices in unforeseen and exciting ways. Expanding access to digital medicine requires integrating technology, operational models, policy, and application scenarios. For example, medical institutions can establish intelligent medical service platforms powered by AI, providing services such as consultation, education, and decision support to meet patients’ diverse needs. Encouraging innovative research to explore these technologies will be vital in leveraging their potential to enhance healthcare delivery and outcomes.

### 3.6 Quantitative privacy risk assessment in Chinese health and medical data

Developing privacy risk assessment tools customized for Chinese health and medical data is crucial for quantifying privacy risks associated with data use. Assessing both data set quality and sensitive information security is essential for minimizing risk and information loss [10]. While studies have shown that the Health Insurance Portability and Accountability Act (HIPAA) policy has a similar protective outcomes in patient data in China [11], further evidence is required to validate its effectiveness in the Chinese context.

Medical multi‐model data storage should be restricted to medical or government institutions. Establishing balanced policies for data use and privacy protection is essential, covering the entire data lifecycle – data generation, transmission, storage, use, sharing, destruction, and management. Technologies such as federated learning can enable data sharing while ensuring data security. These policies and technologies will help create a robust data application ecosystem, ensuring the responsible and ethical use of health and medical data in China.

## 4 COLLABORATIVE INNOVATION ON DIGITAL HEALTH IN CHINA

In summary, the GIDH provides a comprehensive guide for developing digital health in China. Leveraging GIDH's principles and recommendations, we have identified key challenges and proposed solutions tailored to China's healthcare system. It is essential to emphasize the importance of collaborative innovation and stakeholder engagement in digital health. By fostering a culture of collaboration and drawing on the expertise of diverse stakeholders, China can drive impactful and sustainable advancements in digital health strategies and initiatives.

## AUTHOR CONTRIBUTIONS

**Ge Wu**: Visualization (equal); writing—original draft (equal); writing—review and editing (equal). **Mengchun Gong**: Conceptualization (equal); funding acquisition (equal); project administration (equal); writing—original draft (equal); writing—review and editing (equal). **You Wu**: Visualization (equal); writing—review and editing (equal). **Li Liu:** Supervision (equal); writing—review and editing (Supporting). **Boyang Shi**: Visualization (equal). **Zhirong Zeng**: Funding acquisition (equal); project administration (equal); supervision (equal); writing—review and editing (equal).

## CONFLICT OF INTEREST STATEMENT

The authors declare that they have no conflicts of interest.

## ETHICS STATEMENT

Not applicable.

## INFORMED CONSENT

Not applicable.

## Data Availability

This article does not contain research data.
